# Defining early education quality using CLASS-observed teacher-student interaction

**DOI:** 10.3389/fpsyg.2023.1110419

**Published:** 2023-07-14

**Authors:** Robert C. Pianta, Tara Hofkens

**Affiliations:** School of Education and Human Development, University of Virginia, Charlottesville, VA, United States

**Keywords:** teacher-student interactions, early childhood quality, early education policy, preschool quality, teacher effectiveness

## Abstract

In this paper we argue that the quality of early education programs or classrooms can be defined in terms of features of teachers’ interactions with students observed using the Classroom Assessment Scoring System, or CLASS. We present evidence suggesting that dimensions of teacher-student interactions can be described, observed, and measured consistently across cultures and countries and that such dimensions also have modestly positive influence student development and learning. Evidence is summarized indicating that interactions can also be improved systematically through professional development interventions. The paper relies on a framework that describes core features of effective teacher-student interactions present across countries’ highly varied settings and cultural contexts. Limitations of the study include exclusive reliance on the CLASS and that most countries were not low or middle income. We discuss the cross-cultural applicability of the framework and outline suggestions for education policy and practice and future directions for research.

## Introduction

Large-scale studies of educational “inputs” intended to promote student learning (e.g., funding, class size, teacher qualifications) reinforce the inference that students’ experiences in classrooms are the primary agent of their progress (e.g., Nye et al., 2004; Reardon et al., 2013), including in programs serving preschool-age children (Mashburn et al., 2008). This finding is not limited to studies of United States samples but has been reported in preschool and elementary grades from countries across the globe, as varied as Chile, China, and Finland (e.g., Yoshikawa et al., 2015; Virtanen et al., 2018; Hu et al., 2020), in some of which preschool programming tends to be more formalized (UNESCO, 2015; UNICEF, 2019). In efforts to better understand and improve the processes within classrooms for young children that are responsible for these results, teachers’ interactions with students are among the most well-studied and promising elements from among other aspects of classroom experience, including aspects of the physical environment, structural features (e.g., class size), or programmatic elements such as curricula (Mashburn et al., 2008; Fuller et al., 2017). In this paper we discuss findings from studies using the CLASS (Pianta et al., 2008) assessment of teacher-student interaction in United States and non-United States preschool classrooms that suggest defining early education quality in terms of observable features of teacher-student interaction.

Our and others’ research (see Morrison and Connor, 2002; Pianta et al., 2007; Kane et al., 2014; Vernon-Feagans et al., 2019) has generated a set of findings about teacher-student interactions that have implications for approaches to defining, measuring, and improving the impact of early education systems. Although these findings are based largely on data collected from United States and Western European classrooms, recent work in Latin America (e.g., Carneiro et al., 2019) are not inconsistent with these results. The general conclusions are that: (1) teachers are the most potent asset that the education system provides to foster student learning and development (Sabol et al., 2013); (2) qualities of teacher-student interactions that support student engagement and effort, knowledge and thinking, problem-solving and communication skills, and positive relationships with others are the source of these teacher effects (Carneiro et al., 2019; Vernon-Feagans, et al., 2019); (3) these qualities of teachers’ interactions can be observed and measured, and predict multiple aspects of student development (Morrison and Connor, 2002; Vernon-Feagans, et al., 2019); (4) effective interactions can be learned and improved (Hemmeter et al., 2015; Piasta et al., 2015); and (5) supporting effective teacher-student interactions at scale requires workforce development systems that integrate measurement and improvement support (Pianta et al., 2020a).

These conclusions also align with experience accumulated from the implementation of tools to assess and improve teacher-student interactions over the past decade, through which practitioners and policymakers alike describe the capacity created to support student learning when teachers and their interactions with students are made explicit as a developmental and educational resource (Pianta and Allen, 2008; Lemov, 2010; Hemmeter et al., 2015). Importantly, although the evidence for interactions as a key component of effective early education, we acknowledge that contemporary analysis of studies in which multiple aspects of programming are examined, including for example the rigor of instruction or dosage of exposure to content, there is also evidence that these features independently and interactively combine to support children’s learning (Pianta et al., 2020a; Nguyen et al., under review).^1^

A focus on “quality” has been a hallmark of early education policy, programming, and research for over three decades (for example, see McCartney et al., 2007). This focus has persisted as expanded access to quality early education and care features prominently in educational and social policy and human capital improvement in low- and middle-income countries as well (UNESCO, 2015; UNICEF, 2019). Over the years, definitions (and measurements) of quality in early care and education have focused on (1) structural elements of programs such as ratios, length of day, staff qualifications, etc.; (2) physical features of the classroom environment and practices related to safety and health; (3) observed aspects of teacher-student interaction that children experience directly; and (4) indices that aggregate across different indicators, such as Quality Rating and Improvement Systems (QRIS). Assessments of quality can focus on any one or combinations of these wide-ranging elements including: the duration of the school day, teachers’ educational levels, and child-teacher ratio; cleanliness and materials, the daily schedule, or how the setting is arranged; or teachers’ behavior, language, and emotional warmth in the classroom. This cornucopia of constructs and associated measures have rendered the term “quality” challenging to interpret or to adopt as a focus of investment or improvement.

We address the multiple operationalizations of the term quality in two ways. First, we assume that “quality” refers to those features of an educational opportunity that contribute to student learning and development, and that vary across individuals’ educational experience. Efforts to identify and ensure exposure to those features are essential to building an effective system. Notably, regulable factors such as ratio, size, length of day, teacher qualifications, or practices related to safety and health as elements of program design and infrastructure which by policy, are intended to be constant across all programs, classrooms, and enrolled children. As features of design, these are valuable as foundations that assure a set of minimal thresholds for programs (McCartney et al., 2007), some of which, such as ratios or length of day, may foster children’s learning (NICHD ECCRN, 2005). However, we apply the term quality to those elements of program experience that more directly contribute to student learning and that vary considerably; this application of the term quality refers to the ways that regulable factors are implemented. This framing for the use of the term quality, sometimes referred to as “process quality” (National Institute of Child Health and Human Development Early Child Care Research Network, 2005) calls attention to variation in children’s experiences despite consistency in structural indicators. Assessment of such features is most often accomplished through use of different methods of direct observation or teacher/caregiver report.

In this paper we draw from a large data set of observations of classrooms across numerous non-United States countries that used the Classroom Assessment Scoring System (CLASS; Pianta et al., 2008) to extend research from United States samples that describe patterns and features of teacher-student interaction that have common value for student learning and development. The published research from which we draw (see Hofkens et al., in press) includes data collected in countries as varied as Sweden (Castro et al., 2017), rural Ecuador (Carneiro et al., 2019), and China (Hu et al., 2020) to study the nature, quality, and impact of teacher-student interaction across cultures. The use of a common observational measure across countries also affords the opportunity to examine cross-country similarities in teacher-student interactions. Admittedly, the advantage of a common measure for examining quality across contexts is mitigated by the lack of alternative measures of contrasting definitions (e.g., structural features, aspects of the physical setting). As noted above, teacher-student interaction could be assessed through varying forms of direct observation (ratings, frequency counts) or reports by teachers or program leaders, thus the paper is not only limited by framing quality in terms of classroom processes, it is also limited by using only one method to measure those processes.

We agree with the framing for this collection of papers that quality is a multi-faceted term that may have different referent points for varying stakeholders (e.g., parents, educators, community members, students). It is also relevant to note that efforts to build and expand systems of early education and care in low- and middle-income countries globally may find identifying the key regulable foundational features of programs as important as aspects of process quality that are the focus of this paper. In this multi-faceted context we suggest one perspective for defining quality is that of a trained observer focused on teachers’ classroom interactions with students.

## United States studies on quality as defined through observed teacher-student interaction

As noted earlier the term “quality” is often used in very general and abstract ways. Although it has a certain appeal by implying there are ingredients of early education opportunities that yield positive impacts on children; the term “quality” invites wide-ranging interpretations, which can impede efforts to systematically study and improve programs and their support for children, at scale. If, as we conceptualize, the term “quality,” should reflect a direct link between an educational opportunity and its intended outcomes (Pianta et al., 2020a), then at least one component of defining quality should be students’ direct experiences with teachers who engage them in educationally and developmentally salient learning activities.

As just one illustration of this point, we describe results from two studies that each contrasted the predictive strength of differing operationalizations of quality in the United States (Mashburn et al., 2008; Sabol et al., 2013). Many US states use multi-component assessments of several features of early education programs (often structural) as indicators that are then aggregated into a single composite marker of quality (i.e., Quality Rating and Improvement Systems). These reflect the multi-faceted nature of quality. Studies of these composites suggest that they may obscure or omit aspects of the program predictive of students’ learning and development. For example, in one multi-state evaluation of the indicators included in Quality Rating and Improvement System composites (Sabol et al., 2013), observations of the classroom environment, and particularly of teacher-child interactions, were the only indicators that demonstrated significant relations with children’s school readiness. In other studies comparing the predictive value of quality measures – including observations of teacher-student interactions, observations of multiple features of the classroom environment, and a composite quality indicator developed by the National Institute for Early Education Research (NIERR) – higher ratings of teachers’ observed instructional interactions predicted gains in academic readiness and language while greater evidence of teachers’ emotional support was related to lower levels of problem behavior (Mashburn et al., 2008). Across multiple studies, when observations of teachers’ classroom interactions with children are included in models predicting student learning and development that also include other hypothesized indicators of quality (whether aggregated composites or single indicators such as teacher education, class size, etc.), assessments of observed interaction routinely yield significant associations with student outcomes. In yet another example, Ansari and Pianta (2018) used data from the NICHD Study of Early Child Care and Youth Development to examine whether the quality of early education (birth to 54 months) was predictive of children’s learning and development outcomes through 5th grade. A measure of quality was formed from a rating of observed teacher/provider-child interaction in analyses that also included teacher-child ratio, caregiver training and attitudes, etc. Among all indicators of the childcare or preschool structure or experience, observed quality of teacher-student interaction accounted for the greatest variance in students’ later performance.

### Conceptual frameworks for quality and teacher-student interactions

The studies just described provide empirical support for defining quality in terms of observable features of teachers’ classroom interactions. In the sections that follow, we present more detailed discussion and evidence related to one observational assessment of teacher-student interactions, the CLASS, drawing from work in United States and international samples. It should be emphasized that in the context of international studies of early education and care programs, the Early Childhood Environment Rating Scale (ECERS) and its versions for younger children and more recent revisions, have been used much more widely than CLASS (e.g., Vermeer et al., 2016; Betancur et al., 2021) and recently the Measuring Early Learning Environment Scale has shown promise in observations conducted in sub-Saharan Africa (Raikes et al., 2020). In the recent work by Betancur et al. (2021) and Vermeer et al. (2016) analyses revealed teacher-student interactions to be one of only three factors (a more limited set than described in the manual) and, as is typical in observation studies, associations with child outcomes were modest.

A thorough description of theory motivating the CLASS as an indicator for quality is provided in Hamre et al. (2013) presentation of the Teaching Through Interactions (TTI) framework. The TTI framework draws heavily from earlier theoretical and empirical work (e.g., Brophy, 1999; Eccles and Roeser, 2011) and describes a theory for defining, describing, and measuring teachers’ classroom interactions, as operationalized in the CLASS observational tool. The TTI framework organizes teacher-student interactions around three broad domains of teachers’ support for student development – Emotional Supports, Classroom Organization, and Instructional Supports (Hamre et al., 2013; see Table 1). These are based on their presumptive salience for student development in the areas of social and emotional development, self-regulation and attention, and achievement, respectively. Within each of these three broad domains, the TTI specifies a set of dimensions of interaction (e.g., Teacher Sensitivity, Effective Behavior Management, Quality of Feedback) that provides detailed behavioral markers and descriptions of indicators of each dimension as they may appear at low, medium, and high levels. A body of work on teacher-child interactions draws from the TTI framework and the CLASS observational measure (Pianta et al., 2008).

**Table 1:** CLASS framework for early childhood classroom quality.

Area	Dimension	Description
Emotional Support	Positive Climate	Reflects the overall emotional tone of the classroom and the connection between teachers and students
	Negative Climate	Reflects overall level of expressed negativity in the classroom between teachers and students (e.g., anger, aggression, irritability)
	Teacher Sensitivity	Encompasses teachers’ responsivity to students’ needs and awareness of students’ level of academic and emotional functioning
	Regard for Student Perspectives	The degree to which the teacher’s interactions with students and classroom activities place an emphasis on students’ interests, motivations, and points of view, rather than being very teacher-driven
Classroom Management	Behavior Management	Encompasses teachers’ ability to use effective methods to prevent and redirect misbehavior, by presenting clear behavioral expectations and minimizing time spent on behavioral issues
	Productivity	Considers how well teachers manage instructional time and routines so that students have the maximum number of opportunity to learn
	Instructional Learning Formats	The degree to which teachers maximize students’ engagement and ability to learn by providing interesting activities, instruction, centers, and materials
	Classroom Chaos	The degree to which teachers ineffectively manage children in the classroom so that disruption and chaos predominate
	Classroom Management	The degree to which teachers provide clear instructions, rules, and routines that children clearly know and understand, as well as well-timed proactive behavioral strategies rather than control techniques
	Child Responsibility	The extent to which teachers provide children with the opportunity to take on roles and operate autonomously in the classroom
Instructional Support	Concept Development	The degree to which instructional discussions and activities promote students’ higher order thinking skills versus focus on rote and fact-based learning
	Quality of Feedback	Considers teachers’ provision of feedback focused on expanding learning and understanding (formative evaluation), not correctness or the end product (summative evaluation)
	Language Modeling	The quality and amount of teachers’ use of language-stimulation and language-facilitation techniques during individual, small-group, and large-group interactions with children
	Instructional Conversation	Considers the extent to which teachers’ verbal interactions with children are reciprocal and focus on the facilitation of reasoning, concept development, expression of ideas, and cognitive elaboration
	Literacy Instruction	The extent to which teachers reads to children, provides explicit phonics instruction, elaborates on books with comprehension and process questions, and exposes children to written language
	Richness of Instructional Methods	The extent to which teacher use a variety of strategies to promote children’s thinking and understanding of material at deeper and more complex level

Research using the CLASS in United States early education and care settings provides both evidence supporting the three hypothesized domains of interactions in the TTI framework as a theoretically and empirically sound approach to describing and measuring the quality of teacher-student interactions in classroom settings (Hamre et al., 2013), although other studies have pointed to a single overall quality of interactions factor as the most parsimonious descriptor (Pianta et al., 2020a). Results from a study of CLASS observational data from over 4,000 preschools to fifth grade United States classrooms (Hamre et al., 2013) supported the three-domain structure. Analysis of CLASS-based observations in upper elementary and secondary grades from the Measures of Effective Teaching sample of more than 3,000 classrooms (Kane et al., 2014), also affirmed these three broad areas as potentially useful descriptors of teachers’ practices.

In the early education and care sector, studies have also converged on a general picture of the quality of interactions with teachers experienced by the typical preschooler in the United States. Using the CLASS and other observational tools, numerous studies report that quality of teacher-student interaction varies markedly, ranging from sensitive and stimulating, to dismissive and harsh. In the National Center for Early Development and Learning’s study of state prekindergarten programs, only 15 percent of classrooms demonstrated high-quality interactions across 2 of the 3 CLASS domains, whereas 19 percent of classrooms scored well below the mean on emotional, organizational, and instructional supports (Pianta et al., 2005). In general, although the average level of teachers’ emotionally supportive interactions is moderately positive and warm, the picture revealed by observations in thousands of childcare and early childhood classroom settings suggests relatively positive socioemotional and organizational supports, and notably low levels of teachers engaging in stimulating, conceptual conversations or providing rich feedback on students’ learning; for the most part, “teaching” in these settings is highly focused on rote learning of discrete and decontextualized knowledge. Children from low-income families and historically marginalized groups are more likely to experience fewer effective interactions in early childhood programs than their non-poor or privileged peers (Kuhfeld et al., 2019); these findings are not dissimilar to those using other observational protocols in early education settings.

### Teacher-student interactions and student outcomes

Teacher-student interactions are a central element of classroom processes related to children’s learning (Ansari and Pianta, 2018; Vernon-Feagans et al., 2019; Vitiello et al., 2020), whether observed using CLASS, ECERS or other observational systems (e.g., Hemmeter et al., 2015). Learning gains appear to be modestly greater when teachers emphasize conceptual understanding, provide feedback that extends students’ skills, and engage children in conversations (National Institute of Child Health and Human Development Early Child Care Research Network, 2005; Burchinal et al., 2010). Similarly, children whose teachers create an organized and emotionally supportive classroom demonstrate improvements in self-regulatory and social-behavioral outcomes; in fact, children who display problems in self-regulation appear to benefit even more from exposure to effective teacher-child interactions (Hamre and Pianta, 2005; McCartney et al., 2007; Vernon-Feagans et al., 2019). Multiple years of exposure to effective teacher-student interactions appears to be of additional benefit (Cash et al., 2018; Vernon-Feagans et al., 2019), although it is not the norm (Pianta et al., 2007).

Effect sizes obtained between observed features of teachers’ interactive behaviors and student outcomes such as achievement test scores are small (Brock et al., 2008; Mashburn et al., 2008; Rimm-Kaufman et al., 2009; Burchinal et al., 2010; Pakarinen et al., 2011), with larger correlations for students with higher risk profiles (Hamre and Pianta, 2005; McCartney et al., 2007), or for associations with students’ motivation (Ferguson and Hirsch, 2014). Specifically, in United States studies, children who come from low-income families, who are dual language learners, or who have problems with self-regulation appear to benefit more from effective teacher-student interactions than do their more-resourced peers (e.g., Hamre and Pianta, 2005; Desimone and Long, 2010; Ansari et al., 2020). Children reap the most academic benefit from effective teacher-student interactions when they are exposed to such interactions for several years (Cash et al., 2018; Vernon-Feagans et al., 2019).

Most published studies have used statistical controls to reduce or adjust for selection effects. Evidence from recent intervention studies and random assignment studies support a causal link. In experimental evaluations, when teachers improve their practices after they receive training and coaching on teacher-student interactions, the children in their classrooms benefit academically, socially, and behaviorally (Hemmeter et al., 2015; Pianta et al., 2021). Professional development interventions designed to improve teacher-student interaction demonstrate positive impacts of targeted professional development on both teacher-student interaction and student outcomes in preschool and early elementary grades (Boston Consulting Group, 2019; Pianta et al., 2020a). Other evidence for a causal link comes from studies that randomly assigned children to classrooms (Campos et al., 2021). One study conducted in Ecuadorian first- and second-grade classrooms, estimated that teachers in the top 25 percent in terms of the quality of their interactions produced the equivalent of almost 9 months more of achievement growth than did teachers in the bottom 25 percent (Campos et al., 2021).

### Improving interactions and student outcomes through professional development

Tools for observing teachers’ classroom interaction are also a focus for professional development (PD) that targets the interactions defined by those tools. Hemmeter et al. (2013) have used the *Teaching Pyramid Observation Tool* (TPOT; Fox et al., 2014) to guide coaching focused on teachers’ support for children’s social and emotional skills. The TPOT measures a set of practices that promote positive behavior among young children. Coaches implementing Practice-Based Coaching conduct TPOT observations to define targets for their work with teachers; their feedback leads to changes in teachers’ practice (Hemmeter et al., 2013, 2015) and observed improvements in children’s social skills. PD models designed to focus on improving teachers’ interactions based on the CLASS (Pianta et al., 2008) include a college course and a video-based coaching model that have demonstrated positive impacts on teaching practice and, in several studies, on student outcomes (Pianta et al., 2008; Hamre et al., 2012; Pianta et al., 2021). Evaluations of MyTeachingPartner coaching showed that when teachers received MTP coaching, children made greater gains in receptive vocabulary, task orientation, and prosocial assertiveness (Pianta et al., 2021).

## Summary of United States studies

The sections above present evidence from United States studies demonstrating that dimensions of teacher-student interactions can be described, observed, and measured consistently. Studies also indicated that dimensions of teacher-student interaction positively influence student development and learning. Finally, evidence indicates that interactions can be improved systematically through PD interventions. This line of evidence suggests a logic such that interactions could reasonably be considered a focus for describing, defining, measuring, and improving quality in early education classrooms. Below we summarize results from a recent systematic review and meta-analysis drawing from observations of classrooms outside of the United States (Hofkens et al., in press) in an initial effort to examine the extent to which these conclusions from the United States literature may extend more broadly to using observed interactions between teachers and children as a defining feature of early education quality across other countries and cultures.

## International studies on quality as defined through observed teacher-student interaction

Although much of the research using classroom observation (mostly CLASS or ECERS) has been conducted in United States preschool and elementary classrooms, recent work in a variety of international settings—including Central and South America, Europe, and Asia—has also documented that teacher-child interactions support development and learning (e.g., Yoshikawa et al., 2015; Vermeer et al., 2016; Virtanen et al., 2018; Hu et al., 2020; Betancur et al., 2021). Because of broadening focus on the quality of early education in non-United States countries (UNICEF, 2019) and the use of CLASS in studies of these countries’ early education systems, we conducted a systematic review of the published literature reporting data on observed teacher-student interaction from non-United States samples (Hofkens et al., in press).

Hofkens et al. (in press) culled published empirical studies cited in search engines relevant in psychology and education (PsychInfo, ERIC, Google Scholar, Academic Search Complete, Education Research Complete, Education Full Text). They also included in the search databases for masters and dissertations (ProQuest and LIBRA Institutional Repository hosted out of the University of Virginia), websites of documents from large-scale studies that use the CLASS measure (RAND, Measures of Effective Teaching, the National Institute of Child Health and Human Development Study of Early Child Care and Youth Development, and the Early Childhood Longitudinal Study Head Start Impact Study), and the What Works Clearinghouse. Covidence software was used to remove duplicates. Remaining citations were systematically screened (double screened with discrepancies resolved through consensus) using the following criteria. Journal articles, reports, briefs, or theses were retained for further analysis if they reported CLASS data for which: 1) raters were trained using standard approaches and reliability data were included; 2) the sample included at least 20 lead or subject-specific teachers in 3) the classroom was preschool (defined as serving children ages 3–4) or kindergarten (a working definition of “early education”). Thus, reports were not considered further if they focused on infants/toddlers or childcare settings, summer or after school programs, included fewer than 20 teachers, did not include CLASS data, did not report reliabilities for trained observers, or did not present evidence that observers were trained. An author from each document was contacted to request information about other documents that met inclusion criteria and included any new documents in the database. The full database included 365 documents from 133 studies, among which 52 documents were from 19 separate data collection efforts that used the CLASS outside of the United States (Hofkens et al., in press). Notably, most of the countries included in this meta-analysis could be considered middle income and had established policies and program infrastructure for early education.

The 19 studies reported observational data using CLASS in 2,186 separate prekindergarten and kindergarten classrooms (trained raters averaged 3.3 observed cycles over 1.6 days; see Table 2 reproduced from the original Hofkens and colleagues’ paper [in press]). This data set, from the standpoint of stakeholders’ perspectives on early education quality around the globe, enabled us to understand if: (1) raters (as stakeholders) could, after training, agree on a common set of quality features; (2) whether the pattern of those features was similar or different across countries/cultures; and (3) if studies reported them, the extent of associations between teacher-student interaction and children’s learning and development. Below we extend the analysis of Hofkens and colleagues to further elaborate on the CLASS factor structure and its meaning for defining quality, as well as the implications of a common language and lens for quality based on observing interactions.

**Table 2:** Studies of international classrooms that measure teacher-student interactions with the Classroom Assessment Scoring System.^c^

Description						Overall CLASS score			Emotional support mean			Instructional support			Classroom organization		
Citation	Country	# Teachers	# Students	Mean class size	Grade(s)	Mean	SD	ICC (Kappa)	Mean	SD	*α*	Mean	SD	*α*	Mean	SD	*α*
Niklas and Tayler (2018)	Australia	265^a^	2,123	9	Pre-K	4.04^a^	0.93^a^	0.80^a^	5.14	0.91	0.87	2.38	0.96	0.85	4.60	0.92	0.89
Besnard and Letarte (2017)	Canada	53	180	3^a^	Pre-K	4.22^a^	0.69^a^	0.80^a^	5.26	0.72	.	2.59	0.63	.	4.82	0.73	.
Yoshikawa et al. (2015)	Chile	119	1876	21^a^	Pre-K	3.55	.	(0.94)	4.64^a^	.	.	1.72^a^	.	.	4.30^a^	.	.
Hu et al. (2016)	China	180	5,841^a^	32	K	3.98^a^	0.70^a^	0.89^a^	5.03	0.69	0.78	2.12	0.61	0.84	4.80	0.81	0.92
Slot et al. (2018)	Denmark	402	3,132	21	Pre-K	4.66^a^	0.48^a^	0.90^a^	5.85	0.42	0.73	2.45	0.55	0.64	5.69	0.47	0.83
Pakarinen et al. (2010)	Finland	49	679	14	K	4.81	0.79	0.85	5.13	0.80	0.93	3.97	0.92	0.88	5.34	0.66	0.90
Stuck et al. (2016)	Germany	57^a^	390	7^a^	Pre-K	4.44^a^	0.61^a^	0.73	5.57^a^	0.67^a^	0.86	1.63^a^	0.54^a^	0.90	6.13^a^	0.61^a^	0.90
Von Suchodoletz et al. (2014)	Germany	63	1,323^a^	21	Pre-K	4.21^a^	0.85^a^	0.80^b^	5.33^a^	0.75^a^	0.89	2.47^a^	0.78^a^	0.81	4.82^a^	1.02^a^	0.85
Cadima et al. (2018)	Portugal	178	3,827^a^	22	Pre-K	3.81^a^	0.99^a^	0.62	4.48^a^	1.08^a^	0.91	2.27^a^	0.93^a^	0.86	4.67^a^	0.97^a^	0.94
Sandstrom (2012)	Spain	25	634	25	Pre-K	3.86	0.56	.	4.79	0.63	.	2.16	0.49	.	4.32	0.67	.
Castro et al. (2017)	Sweden	165	850^a^	5	Pre-K	5.24	0.95	0.80^b^	5.66	0.74	.	4.76	0.97	.	5.31	1.13	.
Ertürk Kara et al. (2017)	Turkey	120	.		Pre-K	4.05^a^	1.17^a^	0.80^b^	4.11	1.10	0.78	1.90	1.09	0.85	3.36	1.31	0.92
Hoang et al. (2018)	Vietnam	60	1,474	27	K	4.54^a^	1.35^a^	0.78^a^	4.67^a^	1.39^a^	0.88	3.02^a^	1.13^a^	0.95	5.91^a^	1.51^a^	0.91

a Value derived from other data (Class overall mean score: Calculated overall CLASS score from CLASS domain scores; Class domain: calculated with dimension scores; Class size: calculated by dividing the number of teachers/classrooms from the number of students; Students: calculated by multiplying the average class size by the number of classes; ICC: calculated as an average across days and/or aggregated up with domain or dimension-level scores; Teachers: input number of classrooms when teacher information not provided; averages were weighted if from different sized groups).

b Pseudo-ICC calculated from percent agreement.

c Adapted from Hofkens et al. (in press).

### Observing and describing interactions with a common measure across countries

The overall inter-rater reliability across all studies and corresponding CLASS dimensions in Hofkens et al. (in press) paper (reported as intraclass correlations, percent agreement, or kappa scores) was reported as good to excellent (ranging from coefficients of 0.65–0.94), with the exception of one study of Portuguese preschools (Cadima et al., 2014) which had moderate inter-rater reliability (Ranganathan et al., 2017). Furthermore, the internal consistency of CLASS domains appeared consistent across different cultural contexts. More specifically, Hofkens et al. (in press) used reliability generalization as a meta-analytic technique to establish 95% confidence intervals (Rodriguez and Maeda, 2006) for each of the three CLASS domains for the studies in which internal consistency coefficients were reported. The respective confidence intervals for the three CLASS domains (Emotional Support, Instructional Support, Organizational Support) varied between 0.81–0.89; 0.87–0.94; and 0.78–0.87′ respectively, suggesting that the internal reliability for each domain was high across the international studies. These analyses of different indicators of reliability provide preliminary evidence that the TTI framework (as operationalized by CLASS) describes aspects of teacher-student interactions that are evident in classrooms across different cultural contexts.

More specifically, several studies outside the US directly evaluated the 3-domain framework organizing teacher-student interaction (Sandstrom, 2012; Cadima et al., 2014; Gamlem and Munthe, 2014; Besnard and Letarte, 2017; Castro et al., 2017; Gasser et al., 2018; Niklas and Tayler, 2018; Pöysä et al., 2019). These analyses of the factor structure of the CLASS suggest support for the 3-domain framework in early education classrooms across the globe, including prekindergarten samples in Chile (Yoshikawa et al., 2015 as cited in Leyva et al., 2015), Denmark (Slot et al., 2018), and Turkey (Ertürk Kara et al., 2017), and in kindergarten samples in Germany (Von Suchodoletz et al., 2014), Vietnam (Hoang et al., 2018), and in China, where there was also support for a bi-factor model (Hu et al., 2020).

The Negative Climate dimension did not appear to be a significant component of the Emotional Support domain in several countries. In a systematic examination of the CLASS Pakarinen et al. (2010) found that quality of the Finnish kindergarten teachers in their samples was best represented when the Negative Climate dimension was omitted. Similarly, noting the poor discriminate validity of the Negative climate dimension in the previous study, Stuck et al. (2016) also omitted the dimension their study of 57 prekindergarten teachers in Germany. In another study of almost 180 prekindergarten teachers in Portugal, Cadima et al. (2018) found that when they omitted the Negative Climate dimension, the three-factor model provided the best relative fit to the data. It should be noted that contemporary guidance on the use of CLASS in research and in applied implementations suggests excluding Negative Climate from the domain-level computations.

### Quality of teacher-student interaction across countries

Hofkens et al. (in press) reported the first multi-country non-United States view of CLASS-observed teacher-student interaction, albit mostly relying on studies of European or developed countries. Overall, results across this somewhat narrow scope of international studies reflect the American research: mostly mid (4) to middle-high scores (5) for the Emotional Support and the Classroom Organization domains, and lower (2) to low-mid scores (3) for the Instructional Support domain (e.g., Harnes et al., 2014). In this limited international sample, the highest scores are reported in Classroom Organization, with multiple studies reporting a high score (mean level of almost or over 6), which is somewhat higher than in the United States, in which the highest scores are typically associated with the Emotional Support domain, at least in younger-grade samples. Not dissimilar to results from the United States, this multi-national analysis indicates the mean level of Instructional Support is 2.7 across the studies; several studies reported Instructional Support in the low range (1–2), with only a few reporting mid-range scores (3–5). This pattern of low levels on the CLASS Instructional Support domain is consistent with United States findings and suggests that most of the instruction in classrooms across an even broader set of countries focuses on learning discrete facts and skills through instruction that has a rote focus. Adjusting for the reliability among raters in each study (Wiernik and Dahlke, 2020), Hofkens et al. (in press) describe similar findings to those summarized above. The resulting picture of classrooms from this small sample of non-United States classrooms suggest they may be more highly structured, on average, than in the United States, however all samples depict a high degree of variability across classrooms.

### Teacher-student interaction and student outcomes outside the United States

Although the nature and magnitude of the associations between teacher-child interactions and student outcomes varies across these studies, Hofkens et al. (in press) analysis suggests that young students’ quality of interactions with teachers play a modest role in their developmental and academic success. For example, overall quality of interactions is moderately correlated with preschoolers’ attention and impulse control in Turkey (Ertürk Kara et al., 2017), and cognitive self-regulation among socially disadvantaged preschoolers in Portugal (Cadima et al., 2016a), with interaction quality particularly effective in supporting students low in self-regulation skills (Cadima et al., 2016b). For young students in China, instructional support was associated with growth in executive function skills (Hu et al., 2020). In the large longitudinal experimental study in Ecuador, children in grades K-4 who were randomly assigned to teachers who displayed higher quality interactions had higher executive function skills, particularly for working memory (Campos et al., 2021). Higher quality interactions also reduced the likelihood of behavioral problems in the same year (Campos et al., 2021).

Regarding teachers’ interactions that focus on organizational or instructional support of learning opportunities, among a sample of Finnish kindergarten students, the quality of teachers’ instructional support was positively associated with student empathy and negatively associated with disruptive behavior (Siekkinen et al., 2013) and less task avoidant behavior in class (Pakarinen et al., 2011). Furthermore, the quality of teachers’ classroom organization predicted learning motivation among Finnish kindergartners (Pakarinen et al., 2010). And across various cultural settings, teachers’ emotionally supportive interactions, defined by identifying and responding to the emotional needs of their students, also supported student engagement in learning. In Swedish preschools, emotional support predicted student engagement (Castro et al., 2017) and a combination of positive climate, instructional learning formats, and language modeling predicted children’s engagement in literacy learning (Norling et al., 2015).

Finally, each of the three domains of interaction quality predicted students’ academic skills in many of the non-United States samples, including among Danish preschoolers (Slot et al., 2018) and Ecuadorian K-4th grade students, with the strongest effects in kindergarten and first grade (Campos et al., 2021); effects from kindergarten were evident into 6th grade (Campos et al., 2021). In Australia, teachers’ instructional support predicted verbal skills among preschoolers (Niklas and Tayler, 2018), while in China, it is positively associated with reading, math, and science achievement among preschoolers (Hu et al., 2017). Other dimensions of interaction also contribute to academic skill growth. For example, emotional support in kindergarten was also positively associated with Finnish children’s reading skills in first grade (Silinskas et al., 2017) and in Portugal, teachers’ classroom organization predicted first grade students’ vocabulary and print concepts (Cadima et al., 2010).

Together, research from this limited sample of international studies contributes additional empirical support for the teacher-student interactions as a developmentally salient feature of educational settings across cultures. In a combination of large-scale implementations, quasi-experimental, and experimental studies, the quality of teacher-student interactions shows modest associations with developmental and academic outcomes in very different cultural settings.

## Conclusions and implications

In early educational settings, the preponderance of evidence suggests that teacher-student interactions play a significant role in fostering students’ development and learning across wide-ranging countries and cultures; and as we have reported, from United States studies, interactions are responsive to targeted improvement models such as coaching. For these reasons, describing, measuring, and improving teacher-student interactions, as a key feature of “quality” could be helpful to large-scale efforts to build and improve public education systems (Pianta and Hamre, 2022). The present study is an effort to examine parallels from non-United States samples to the larger evidence base from United States studies to examine the extent to which there is consistency in findings on teachers and students in non-United States countries across the globe.

By and large the results obtained from the United States and a multinational synthesis are quite consistent. Across the 16 countries, 4,400 teachers, and 42,000 students included in Hofkens et al. (in press) review and meta-analysis, the following conclusions were supported: (1) teacher-student interactions can be describing using a common set of descriptors and reliably observed using those descriptors across countries that vary in cultural and educational circumstances; (2) teacher-student interactions in United States and non-United States samples appear to have a common latent structure or organization such that aspects of teachers’ emotional, instructional, and organizational behavior align with a framework for description that can be used consistently across countries; (3) these three broad domains of interaction have significant and beneficial impacts on students’ learning and development. Although with many fewer exemplars (e.g., Yoshikawa et al., 2015), international studies also report that these common features of interaction can be improved through focused training and supports. Collectively, this pattern of results has powerful implications for theories of educational processes, for investments in workforce development systems that define quality in terms of observed interaction, and for professional development efforts that focus on teacher-student interaction as a means to improve the quality of educational opportunity and outcomes (Pianta and Hamre, 2022).

The conclusions above should be framed by certain caveats and limitations. The most notable among these qualifications is the limited variability in the Hofkens et al. international data set. The studies included in that analysis largely reflect Western approaches to early education in middle-upper income countries with far fewer low- and middle-income countries and cultures than would support a truly globalized international perspective. The CLASS was used as a common classroom observation tool to capture general properties of classroom interactions, without modifications to reflect nuances unique to culture, ethnicity, race, or language. A more recent edition of the CLASS (Teachstone, 2022) explicitly acknowledges cultural differences and nuanced interpretations of teacher-student interactions and may be better-suited for cross-cultural and cross-national work. As acknowledged earlier, the use of the CLASS across these wide-ranging settings is both an advantage and a disadvantage for examining evidence for a common definition. That is, a common metric is essential to analysis of consistency across varying contexts, while the lack of alternatives (either metrics of teacher-student interaction or of competing definitions of quality) constrains the interpretations that can be made, pointing to the need for further systematic research.

As a further limitation, the descriptive statistics reported (e.g., means, variance) in the study of Hofkens et al. (in press) and in the United States studies are all drawn from convenience samples; none are representative of the countries’ populations or school systems. Therefore, cross-country comparisons of these indicators are not advised, nor is it appropriate to draw conclusions about the level of quality of teacher-student interaction in a given country.

That said, the descriptive findings point to the potential use of observations, such as CLASS or other scalable measures, in representative samples of countries or important political, geographic, or cultural groups, which might drive investments in education systems and teacher development. Recent evidence supports framing quality in terms of a “package” of elements that each features observations of teacher behavior and classroom practice: teacher-student interactions, teachers’ exposure of students to content through use of a targeted curriculum, and how teachers individualize their instruction to students’ skills. These all rest on a core of teachers’ knowledge and skill in engaging individual students through relationships and interaction. In a recent investigation, the elements of this package was each independently and additively predicted children’s learning, were uncorrelated, and yielded a larger effect size than each individually (Pianta et al., 2020b).

We are interested in expanding and deepening a theory of teacher-student relationships and their value, as a basis for building and disseminating usable tools and knowledge. Developmental systems theory and attachment theory informed the core of all CLASS dimensions (rating scales) around properties of “serve and volley” exchanges that required attention to both the teacher’s behavior *and* the student’s response. This theory of classroom processes, the *Teaching Through Interactions* framework (Pianta and Hamre, 2009), hypothesized a taxonomic organization and definition(s) of teacher-student interaction that has proven useful in understanding and improving the impact of educational experiences in many thousands of classrooms across the United States and in in non-United States samples as described in this paper. Theory predicted that this latent structure would apply across all grade levels, content areas, or focus of instruction -- that “good teaching is good teaching” across the many permutations in which it takes place, precisely because interactions are a key pathway through which students learn.

With these general conclusions in mind, there are several implications for further cross-national research. Assuming an aim to use a common observational tool across countries, questions of interest might involve the extent to which characteristics of observers (e.g., prior knowledge, cultural background or differences, experience) and their training are associated with differential levels of reliability in the form of agreement. These questions essentially focus on the conditions that may limit or support the use of a common observational tool for defining quality across wide-ranging cultures. Also, as noted earlier, it is essential to expand the evaluation of assessments of quality across a wider range of income and culture, and to include a wider range of potential constricts and metrics that may be more salient in such contexts (e.g., Vermeer et al., 2016; Raikes et al., 2020; Betancur et al., 2021). Looking ahead, we are intrigued by technology (natural language processing, artificial intelligence) that can make observational tools more efficient in terms of time and expense, and more effective. Even if using common too(s)l might be advisable, examining common and country/culture specific features of interaction that foster students’ learning and development might inform observational systems best suited to a culture’s uniqueness as well as capturing what common elements of effective teaching. Research on conceptualizations and measurement tools that define quality in terms of observed interaction, examining the commonalities and differences across countries, cultures, and groups, could help advance and deepen the impacts of interactions and relationships as the core educational resource for students’ learning and development.

## Author contributions

RP and TH made substantial contributions to the manuscript. TH was responsible for carrying out meta-analytic and narrative reviews while RP contributed to manuscript preparation and prior research. All authors contributed to the article and approved the submitted version.

## Funding

The research reported here was supported by the Institute of Education Sciences, U.S. Department of Education, through Grant #R305N160021 to the University of Virginia. The opinions expressed are those of the authors and do not represent views of the Institute or the U.S. Department of Education.

## Conflict of interest

RP is also the author of the Classroom Assessment Scoring System (CLASS), which is a focus of the manuscript. RP is also co-founder of Teachstone, the company that disseminates the CLASS.

The remaining author declares that the research was conducted in the absence of any commercial or financial relationships that could be construed as a potential conflict of interest.

## Publisher’s note

All claims expressed in this article are solely those of the authors and do not necessarily represent those of their affiliated organizations, or those of the publisher, the editors and the reviewers. Any product that may be evaluated in this article, or claim that may be made by its manufacturer, is not guaranteed or endorsed by the publisher.

## Footnotes

^1^Nguyen, T., Pianta, R. C., Whittaker, J. V., Vitiello, V. E., and Ruzek, E. A. (under review). Associations between classroom processes and students’ academic outcomes from pre-kindergarten through first grade. *Elem. Sch. J.*
